# Evaluation of the Effect of Selected Brominated Flame Retardants on Human Serum Albumin and Human Erythrocyte Membrane Proteins

**DOI:** 10.3390/ijms21113926

**Published:** 2020-05-30

**Authors:** Monika Jarosiewicz, Katarzyna Miłowska, Anita Krokosz, Bożena Bukowska

**Affiliations:** 1 Department of Biophysics of Environmental Pollution, Faculty of Biology and Environmental Protection, University of Lodz, Pomorska 141/143, 90-236 Lodz, Poland; anita.krokosz@biol.uni.lodz.pl (A.K.); bozena.bukowska@biol.uni.lodz.pl (B.B.); 2 Department of Cytobiochemistry, Faculty of Biology and Environmental Protection, University of Lodz, Pomorska 141/143, 90-236 Lodz, Poland; 3 Department of General Biophysics, Faculty of Biology and Environmental Protection, University of Lodz, Pomorska 141/143, 90-236 Lodz, Poland; katarzyna.milowska@biol.uni.lodz.pl

**Keywords:** brominated flame retardants, human serum albumin, circular dichroism, tryptophan fluorescence

## Abstract

Brominated flame retardants (BFRs) have been using to reduce the flammability of plastics contained in many products, such as household articles, furniture, mattresses, textiles or insulation. Considering the fact that these compounds may be released into the environment leading to the exposure of living organisms, it is necessary to study their possible effects and mechanisms of action. Proteins play a crucial role in all biological processes. For this reason, a simple model of human serum albumin (HSA) was chosen to study the mechanism of BFRs’ effect on proteins. The study determined interactions between selected BFRs, i.e., tetrabromobisphenol A (TBBPA), tetrabromobisphenol S (TBBPS), 2,4-dibromophenol (2,4-DBP), 2,4,6-tribromophenol (2,4,6-TBP) and pentabromophenol (PBP), and HSA by measurement of fluorescence of intrinsic tryptophan and absorbance of circular dichroism (CD). In addition, in order to understand the possible effect of these compounds in their native environment, the effect of BFRs on membrane proteins of human erythrocytes (red blood cells, RBCs) was also assessed. Among bromophenols, PBP had the strongest oxidative effect on RBC membrane, and 2,4-DBP demonstrated the weakest fluorescence-quenching effect of both membrane tryptophan and HSA. By contrast to PBP, 2,4-DBP and 2,4,6-TBP caused spatial changes of HSA. We have observed that among all analyzed BFRs, TBBPA caused the strongest oxidation of RBC membrane proteins and the model HSA protein, causing reduction of fluorescence of tryptophan contained in them. TBBPA also changed albumin conformation properties, leading to impairment of the α-helix structure. However, TBBPS had the weakest oxidative effect on proteins among studied BFRs and did not affect the secondary structure of HSA.

## 1. Introduction

Brominated flame retardants (BFRs) are the compounds used to reduce flammability and/or retard combustion of organic materials, mostly of polymerized plastics. Despite increasing regulations and a ban on the use of selected BFRs, such as pentabrominated diphenylethers (PentaBDE), octabrominated diphenylethers (OctaBDE), decabrominated diphenylethers (DekaBDE) and hexabromocyclododecane (HBCD), the compounds of this family are still widely used on the market. 

BFRs are commonly used to prevent fires in electronic and electrical equipment, which accounts for more than 50% of their applications. In addition, BFRs are used to reduce the flammability of plastics in many products, such as household articles, furniture, mattresses, textiles or insulation [1].

Tetrabromobisphenol A (TBBPA) has the largest share in the production of BFRs (approximately 60%). Based on in vitro studies it has been determined that this compound may be potentially harmful for the human organism. Due to the fact that TBBPA structure is similar to the structure of the thyroid hormone, thyroxine (T4), and it is a derivative of another endocrine disruptor, bisphenol A (BPA), it is suspected that TBBPA may affect the hormonal system of living organisms, including humans [2]. It is believed that TBBPA may be involved in development of many diseases, including diabetes or cancer [3,4,5,6]. Recent reports have indicated relationships between BFRs and neurodegenerative diseases, in which protein damage (mainly changes in protein spatial structure leading to aggregation and, among-others, formation of β-amyloid in Alzheimer’s disease) play an essential role [7,8]. Al-Mousa (2012) and Michelangeli (2019) have suggested that BFRs at micromolar concentrations caused β-amyloid peptide (Aβ-42) formation and release from human neuroblastoma cells (SH-SY5Y) within a few hours of exposure. Therefore, these results showed that these toxicants are both neurotoxic and amyloidogenic in vitro. 

Our previous studies have demonstrated that BFRs, such as 2,4-dibromophenol (2,4-DBP), 2,4,6-tribromophenol (2,4,6-TBP), pentabromophenol (PBP), tetrabromobisphenol S (TBBPS), tetrabromobisphenol A (TBBPA) at very low concentrations caused an increase of reactive oxygen species (ROS) production [9], altered the activity of the antioxidative system [10] as well as inducing suicidal cell death (eryptosis) [9] and hemolysis in erythrocytes (red blood cells, RBCs) [11].

Many reports have indicated that TBBPS could be a less toxic substitute for TBBPA. However, the latest scientific report indicated that TBBPS was also characterized by harmful activity, e.g., influencing the human neural stem cells’ (hNSCs) ability to self-renew and differentiate, which could account for neural developmental toxicity [12]. TBBPS was also shown to affect the development of the human central nervous system through disturbance of neural ectoderm generation, by dysregulation of expression of multiple genes [13]. 

Proteins play a crucial role in all biological processes. They are catalysts, and are responsible for transport and storage of various molecules, play various mechanical, structural and signal functions, and much more. The tertiary and quaternary structure of proteins, or the spatial arrangement of the polypeptide chain and relative positions and interferences between protein units, influence physical and chemical properties of a molecule and its biological functions. Amino acid composition, or the primary structure, plays the decisive role in conformation of a protein molecule, by formation of hydrogen bonds in the polypeptide chain, leading to the formation of zones of, among others, alpha-helix and beta-sheet. Further interactions of side amino acid residues in various regions of the polypeptide molecule through hydrogen bonds, disulphide bridges, van der Waals forces, and bipolar interactions, lead to formation of the tertiary structure of the protein unit, the mere. Functional proteins are made of one to four units (meres). Relative arrangement of units forms the quaternary structure. Strength of bonds responsible for maintenance of a given structure decreases with increasing order of the structure. Proteins undergo conformational changes upon ligand binding [14]. Those changes are associated with functions played by proteins. However, changes in the spatial structure of a protein under the influence of many exogenous factors, e.g., xenobiotics or ionizing radiation, may also lead to deformation of the protein structure and loss of its biological activity. This, in turn, may result in loss of their function and ultimately translate into pathological conditions in the cell [15]. Numerous studies have demonstrated that xenobiotics and their metabolites cause oxidative modifications of proteins through generation of ROS and reactive nitrogen species (RNS). Oxidative modifications of proteins may result in their aggregation, leading to loss of their physiological function and development of pathologies. Considering their increased production or reduced elimination, accumulation of oxidatively modified proteins is associated with both ageing, and numerous diseases, including neurodegenerative ones [16,17,18]. Moreover, different oxidants generate a broad and sometimes characteristic, spectrum of post-translational protein modifications [19]. 

Taking the above into consideration, it is important to determine the effects of BFRs on proteins, because changes in those macromolecules may be involved in the development of numerous diseases. 

**Scheme 1:**
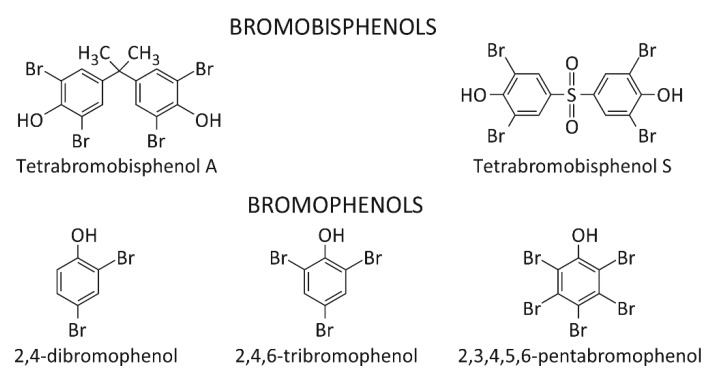
The chemical formulas of brominated flame retardants (BFRs) studied.

A simple model of human serum albumin (HSA) was chosen to study the mechanism of BFRs’ action on proteins. HSA was selected as a model protein with a well-known structure, possessing one tryptophan residue in the subdomain IIA, which simplifies the analysis of fluorescence results. The protein also plays an important role in the process of detoxification of xenobiotics. It has been proven that transport, distribution and final neutralization of xenobiotics entering the organism often depend on HSA, as the best-known carrier protein in the cardiovascular system. Moreover, it is the major circulating antioxidant, able to scavenge large amounts of ROS and RNS [20]. For those reasons HSA seems to be a perfect model for studies of basic ligand–protein interactions. This study assessed the interactions between selected BFRs, i.e., TBBPA, TBBPS, 2,4-DBP, 2,4,6-TBP and PBP (Scheme 1), and HSA by measurement of intrinsic tryptophan fluorescence and evaluating the secondary structure of HSA using the method of circular dichroism (CD). 

Potential binding of BFRs to proteins or their indirect action on protein molecules, e.g., by ROS production, may cause structural and functional changes in proteins, and this may explain some of the pathological conditions observed in chronic BFR exposure. Therefore, for the first time, we have compared the effect of five BFRs on human RBC membrane proteins by measurement of tryptophan fluorescence they contain as well as on HSA. This study allowed us to present the effect of BFRs under native conditions and answer the question as to whether tested compounds were able to damage tryptophan residues contained in proteins of RBC lipid bilayer (native system) and in the chemical system consisting of only the HSA.

## 2. Results

### 2.1. Human Serum Albumin (HSA) Fluorescence

**Figure 1:**
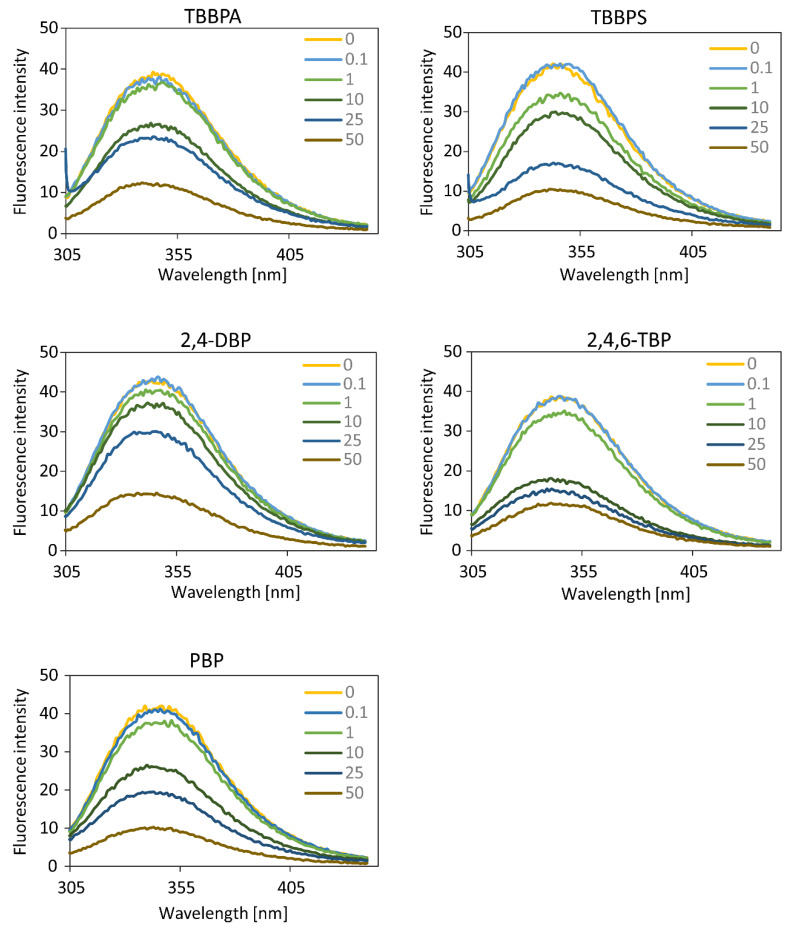
Human serum albumin (HSA, 5 µM) tryptophan residue quenching by tetrabromobisphenol A (TBBPA), tetrabromobisphenol S (TBBPS), 2,4-dibromophenol (2,4-DBP), 2,4,6-tribromophenol (2,4,6-TBP) and pentabromophenol (PBP) at the concentrations ranging from 0.1 to 50 μg/mL.; λexc = 290 nm.

It was found that analyzed compounds caused quenching of tryptophan fluorescence in HSA. Fluorescence loss with increasing concentration of analyzed BFRs was observed (Figure 1), while the maximum of fluorescence did not change.

Then, using the correlation between F_0_/F and the level of the quencher [Q], a Stern–Volmer graph was drawn for low (micromolar) concentrations of the studied compounds, which is recommended for this type of analysis. 

**Figure 2:**
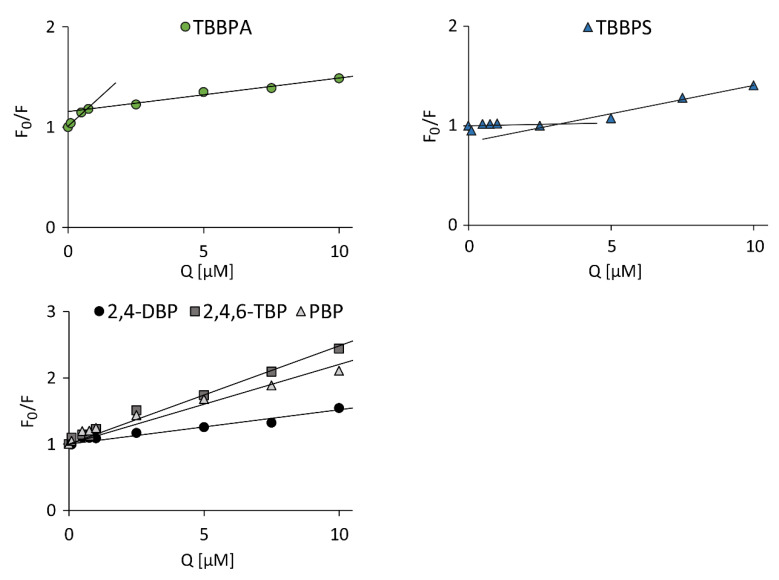
Stern–Volmer plots for HSA-tryptophan fluorescence quenching by analyzed BFRs.

It was observed that tryptophan fluorescence quenching caused by three bromophenols (2,4-DBP, 2,4,6-TBP, PBP) was similar, evidenced by linearity of the plot. However, a non-uniform quenching was observed for TBBPA and TBBPS. Stern–Volmer plots for different BFRs are shown in Figure 2 and Stern–Volmer constants are given in Table 1. Data was analyzed using the Stern–Volmer equation:

F_0_/F = 1+ K_SV_[Q], (1)

where F_0_ and F were fluorescence intensities in the absence and presence of quencher, Ksv was the Stern–Volmer constant or fluorescence quenching constant and [Q] is concentration of the quencher.

Among analyzed bromophenols, the most pronounced loss of fluorescence, and thus the highest increase of the F_0_/F coefficient (and a statistically significant deviation from the directional coefficient “a” of a straight line) was noted for 2,4,6-TBP (0.1385 ± 0.0051, *p* < 0.001), followed by PBP (0.1054 ± 0.0042, *p* < 0.001), and finally by 2,4-DBP (0.0470 ± 0.0031, *p* < 0.001) (Figure 2). 

### 2.2. Circular Dichroism (CD)

**Figure 3:**
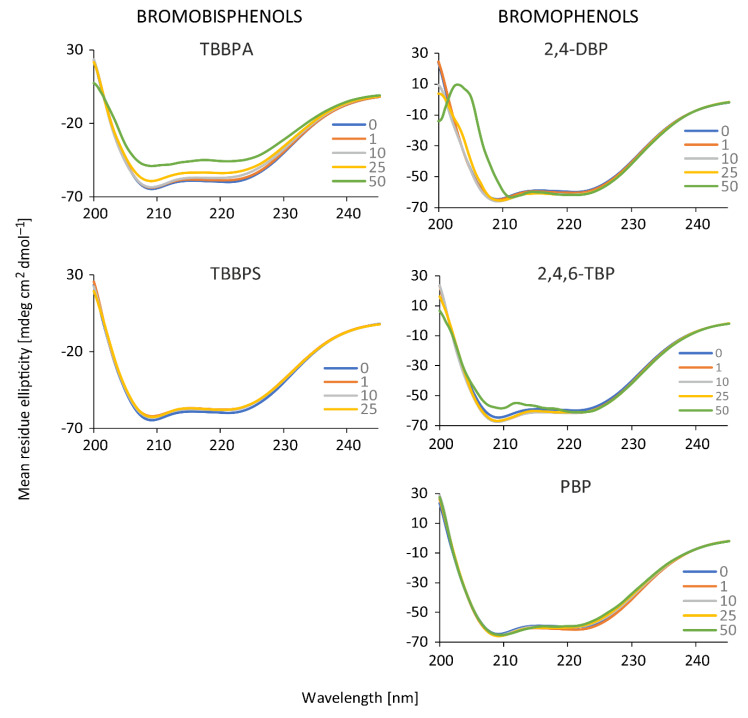
Circular dichroism (CD) spectra of HSA (1 µM) treated with TBBPA (1–50 µg/mL), TBBPS (1–25 µg/mL) and 2,4-DBP, 2,4,6-TBP, PBP (1–50 µg/mL).

It was observed that TBBPA was the only compound causing statistically significant changes in HSA structure even at the concentration of 25 µg/mL. However, at the highest applied concentration of 50 µg/mL three compounds, namely TBBPA, 2,4-DBP and 2,4,6-TBP caused changes in the CD HSA spectrum (Figure 3). TBBPS (1 to 25 µg/mL) and PBP (1 to 50 µg/mL) caused no changes in the secondary structure of HSA. 

It was observed that TBBPA, 2,4-DBP and 2,4,6-TBP caused a decrease in the amount of the α-helix structure from 70.73% of the control sample to 52.30%, 54.53% and 65.40%, for the highest concentration of 50 µg/mL of TBBPA, 2,4-DBP and 2,4,6-TBP, respectively. A minor increase of the ß-sheet structure was also observed in the secondary structure of the protein (from 11.43% for the control sample to 13.97%, 12.90% and 12.03% for TBBPA, 2,4,-DCP and 2,4,6-TBP, respectively). A significant increase of random coil forms was also found for TBBPA and 2,4-DBP, to approximately 22% (*p* < 0.01), and for 2,4,6-TBP to 15.33% (*p* < 0.05), compared to 13.03% observed for the control sample (Table 2). 

### 2.3. Protein Damage

**Figure 4:**
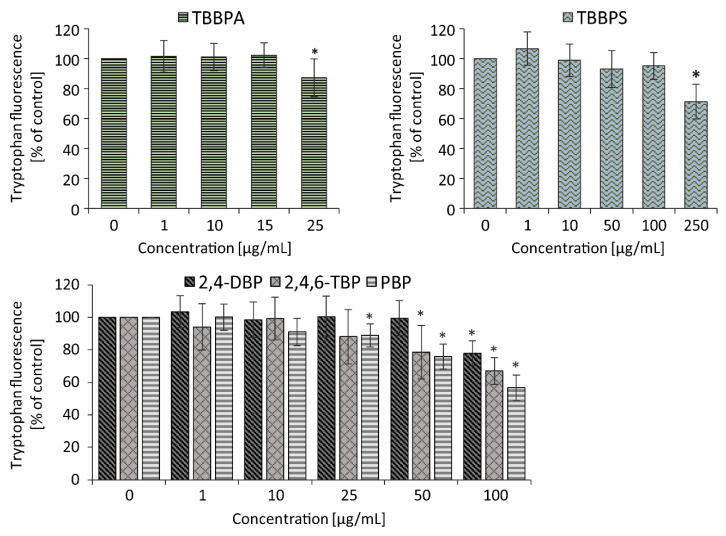
Changes in tryptophan fluorescence (protein damage) in human control red blood cells (RBCs) and the RBCs incubated with TBBPA, TBBPS, 2,4-DBP, 2,4,6-TBP and PBP at the concentrations ranging from 1 to 250 μg/mL for 48 h. (*) Significantly different from control (*p* < 0.05); one-way analysis of variance (ANOVA) and a posteriori Tukey test.

A decrease in tryptophan fluorescence in RBC membranes incubated with analyzed BFRs, compared to control samples, was observed after 48 h of incubation. The highest decrease was noted for TBBPA and PBP (from the concentration of 25 µg/mL). Other bromophenols from 50 µg/mL caused a statistically significant reduction of fluorescence intensity, while TBBPS from 250 µg/mL decreased the studied parameter (Figure 4).

## 3. Discussion

Protein damage may be the result of a direct interaction with toxicants, or an indirect effect of ROS [21,22]. Reactions of ROS with protein lead to modifications of amino acid moieties, modification of prosthetic groups, or to aggregation or fragmentation of protein molecules. All those modifications usually coexist, and their relative share depends on source of ROS and exposure conditions. Most often, the hydroxide radical is the mediator of ROS-induced protein damage. However, some reactions leading to modification of protein molecules, such as oxidation of -SH groups, may be directly triggered by the superoxide anion radical and hydrogen peroxide. ROS may also react with metal ions in metalloproteins [22]. Our previous study [9] demonstrated that analyzed BFRs even at relatively low (environmental relevant) concentrations, caused ROS production in human RBCs. BFRs studied also increased the methemoglobin level in human RBCs [11].

Fluorescence spectroscopy is a very efficient method for studying intramolecular interactions involving proteins [23]. Proteins exhibit fluorescent properties because of the presence of aromatic amino acids, i.e., phenylalanine, tyrosine and tryptophan in their polypeptide chain. Tryptophan is the dominant fluorophore, due to the fact that phenylalanine has a very low quantum efficiency, while the fluorescence of tyrosine may be almost completely suppressed by the use of excitation with the wavelength of 290–305 nm [24,25].

In order to assess the effect of BFRs on protein in a chemical system, we have compared the effect of TBBPA, TBBPS, 2,4-DBP, 2,4,6-TBP and PBP on HSA. This type of study has been repeatedly conducted for drug–protein interaction [26], but very little attention has been paid so far to assess the interactions between proteins, such as HSA and toxic substances. 

The studied compounds caused a concentration-dependent, significant and consistent fluorescence quenching of HSA, which was the most probably due to high level of BFRs–protein interaction. However, BFRs did not cause a shift of emission maximum of HSA, which was centered at 342 nm. The results obtained suggested that the analyzed compounds did not alter the polarity in the vicinity of the fluorophores, especially in the vicinity of the only Trp-214 residue. It is important because Trp-214 is situated in the region of Sudlow’s site, which plays a crucial role in drugs binding [26,27,28]. Molecular modeling studies of Wang et al. [29] proved that TBBPA entered into the center of HSA and located near to subdomain IIA and Trp-214 residue. Other amino acids concerning Ala-191, Lys-195, 199, 436, Leu-198, Arg-218, 222, Asp-451, Val-455 and fluorophore Tyr-452 residues were involved in the interactions of HSA with TBBPA. Additionally, we observed that the Stern–Volmer plot had a linear correlation for bromophenols over the applied range of their concentrations, which indicated a uniform character of fluorescence quenching. The 2,4-DBP had the weakest effect on HSA, and caused the smallest loss of tryptophan fluorescence. 

However, in the case of TBBPA and TBBPS, the Stern–Volmer plot deviated from the x axis, and lost its linearity with increasing BFRs concentration, which could suggest the presence of two fluorophore populations in that system. TBBPA at lower concentrations (0.1–1 µM) caused a significant quenching of tryptophan fluorescence in HSA, while no such effect was observed for TBBPS (0.1–2.5 µM). The Stern–Volmer constant (K_SV_) plot was linear for HSA incubated with higher concentrations of TBBPA and TBBPS. Analysis of deviation of the directional coefficient “a” for TBBPA (0.0339 ± 0.0125) and for TBBPS (0.05691 ± 0.0065) showed no statistically significant differences (*p* = 0.119) for tryptophan fluorescence quenching intensity at higher concentrations (1–10 µM, and 2.5–10 µM, respectively) of these BFRs. If the Stern–Volmer plot is not linear, a higher complexity of the fluorophore quenching process may be discussed. At higher concentrations of both TBBPA and TBBPS, a static quenching may have become significant, showing the formation of a non-fluorescent complex with the quencher, or “instantaneous” adsorption of the emitted energy by a nearby (the Van der Waals radius) molecule of a quencher. The differences in the affinity and binding to HSA of the compounds studied are largely related to their molecular structure. Wang et al. [29] found that TBBPA with 4 bromine atoms binds much more efficiently to HSA than deca-brominated-diphenyl ether (deca-BDE), despite the fact that the second one contains as many as 10 bromine atoms. The large π–π* transitions of the benzene rings in TBBPA could increase the binding ability of TBBPA with HSA [29]. Thus, bromine atoms on the benzene rings enhanced the hydrophobic ability of BFRs. On the other hand, two hydroxyl groups attached to the aromatic rings of TBBPA may create hydrogen bonds with HSA. In addition, the aromatic rings enhance the possibility of the stacking interactions with HSA [29]. Hydrophobic, van der Waals and electrostatic interactions and hydrogen bonds are the main noncovalent binding forces of small organic molecules with proteins [30]. 

A significant quenching of tryptophan in HSA under influence of the highest concentrations of BFRs indicates existence of disorders in the protein conformational structure [25]. CD spectra determining the structure of the protein were acquired in order to verify what type of conformational changes occurred. Measurement of CD spectra are broadly used for determination of the secondary structure, conformation and stability of proteins in solutions. Due to their significant optical activity over the peptide bond absorption band (160–240 nm) those molecules demonstrate a strong and differentiated CD signal, dependent on the accepted secondary structure. Considering the fact that spectroscopic techniques are non-destructive, they may provide valuable information on conformation transitions. It was found that TBBPA, 2,4-DBP and 2,4,6-TBP at their highest concentrations of 50 µg/mL caused changes in HSA spectra (Figure 4). CD spectra of native albumins demonstrate two negative peaks in far ultraviolet (UV) at 208 nm and 222–223 nm, which corresponds to the α-helix structure of the protein [25]. These peaks come from the π —π* and n—π* transitions, respectively, in the polypeptide backbone of HSA [31]. Therefore, it may be concluded that studied compounds altered the secondary structure of albumin, mainly by disrupting the α-helix structure. The course of CD spectra allows determination of the percentage share of individual spatial forms in the total protein structure (Table 2). We observed a decrease in the α-helix structure, as well as a slight increase in the ß-sheet structure and a significant increase in random coil, which suggests that TBBPA, 2,4-DBP and 2,4,6-TBP could affect the secondary structure of HSA, and thus the physiological function of protein, which is consistent with the study of Wang et al., 2014 regarding TBBPA [29]. 

No TBBPS and PBP-induced changes of spatial structure of HSA were observed. Similarly, Wang et al. (2014) showed that BPS (the analogue of TBBPS containing no bromine in its structure) did not induce changes in the secondary structure of bovine serum albumin (BSA) [32].

The 2,4-DBP having the lowest number of bromines caused the most pronounced disorders of the secondary structure of HSA. Probably, the smallest molecule of 2,4-DBP was able to efficiently penetrate the protein and combine with hydrophobic pockets of the II A subdomain, thus disrupting the α-helix structure of HSA. 

Considering the fact that BFRs are potentially able to be incorporated into RBC membrane, triggering eryptosis and hemolysis [9,11], the decision was made to analyze how they can directly affect basic (protein) membrane components. For this purpose, tryptophan fluorescence was measured in the membrane of RBC. 

In this study, it was observed that analyzed BFRs damaged proteins in the erythrocytes membrane, which was observed as a decrease in tryptophan fluorescence. The most pronounced changes were observed in cells treated with TBBPA and PBP from the concentration of 25 µg/mL (approximately 88% compared to the control), while the smallest in RBCs incubated with TBBPS from 250 µg/mL (approximately 71.31% compared to the control). Other BFRs studied from the concentration of 50 µg/mL caused a statistically significant loss of tryptophan fluorescence. Moreover, a correlation was found between increasing number of bromine atoms within bromophenols and reduction of tryptophan fluorescence in the native environment. 

Maćczak et al. (2017) found that bisphenol A (BPA) at 25 µg/mL and bisphenol S (BPS) at much higher concentration of 100 µg/mL caused 65% loss of tryptophan fluorescence [33]. It may be concluded that BPA and BPS at the same concentrations as their brominated analogues (TBBPA and TBBPS) caused a greater decrease in the fluorescence of tryptophan contained in RBC membrane. Perhaps this is due to lower molar mass of bisphenols than their brominated analogs (more than twice the number of BPA and BPS molecules exist than in TBBPA and TBBPS, respectively) at the same concentration (25 µg/mL).

## 4. Materials and Methods

### 4.1. Chemicals

TBBPA (purity 99%, 2,6-dibromo-4-[2-(3,5-dibromo-4-hydroxyphenyl)propan-2-yl]phenol)), 2,4-DBP (purity 98%, 2,4-dibromophenol), PBP (purity 98%, IUPAC name: 2,3,4,5,6-pentabromophenol) were bought from LGC Standards (Germany). 2,4,6-TBP (purity 100%, 2,4,6-tribromophenol) and human serum albumin (HSA, ≥ 96%) were purchased from Sigma-Aldrich, Merck. Tetrabromobisphenol S (purity 98.8%, 2,6-dibromo-4-(3,5-dibromo-4-hydroxyphenyl)sulfonylphenol) was synthetized in the Institute of Industrial Organic Chemistry in Warsaw, Poland. The compounds were dissolved in in dimethyl sulfoxide (DMSO, ≥99.5%, bought from Sigma-Aldrich, Merck, Kenilworth, NJ, USA) to the final concentration of 0.4%. 

### 4.2. Fluorescence Quenching of HSA

HSA was dissolved in PBS at a concentration of 5 µmol/L. The emission spectra were recorded from 300 to 440 nm after excitation at 295 nm with the maximum observed at 342 nm. The width of the excitation and emission slit was 5–10 nm. The measurement was carried out at room temperature (21 °C). BFRs in the concentrations from 0.1 to 50 µg/mL were added to the HSA solution, and fluorescence spectra were recorded (Cary Eclipse, Varian). In addition, low micromolar concentrations of analyzed compounds (0.1–10 µM) were used to evaluate the mechanism of tryptophan fluorescence quenching and to plot Stern–Volmer curves. Control samples with and without DMSO were tested in order to exclude the possibility of fluorescence quenching by the solvent. 

### 4.3. Circular Dichroism

Circular dichroism (CD) is a valuable technique for examining the structure of proteins in solution. CD refers to the differential absorption of 2 polarised components of equal magnitude, one rotating counter-clockwise (left-handed, L) and the other clockwise (right-handed, R) [31]. CD spectra were recorded with the Spectrometer Jasco J-815. A cuvette of 0.5 cm path length was used, and spectra were collected from 200 to 260 nm with spectral bandwidth of 5 nm. Each spectrum was the average of three scans. The measurement was carried out at room temperature (21 °C). HSA was dissolved (1 µmol/L) in phosphate buffer, pH 7.4, which was used as the running buffer. Different concentrations of BFRs ranging from 1 to 50 µg/mL were used (except for TBBPS used at the concentrations ranging from 1 to 25 µg/mL, because of its low solubility in ethanol). 

Since it was observed that DMSO impaired measurement of CD spectra, the compounds were dissolved in ethanol. Spectral data was expressed as the mean residue ellipticity (MRE). Protein secondary structure was evaluated using CD deconvolution.

### 4.4. Erythrocyte and Erythrocyte’s Membrane Isolation

RBCs were isolated from buffy coat obtained from blood from healthy donors from the Regional Centre of Blood Donation and Blood Treatment (Lodz, Poland). The RBCs’ isolation procedure was previously described by Jarosiewicz et al. (2017) [11]. RBCs with hematocrit of 5% (about 630 mln cells/mL) were incubated with tested compounds at 37 °C for 48 h. The samples that contained the RBCs, Ringer buffer and DMSO (final concentration 0.4%) were used as negative controls and compared to the samples without DMSO, so it was possible to exclude its influence on RBCs. RBC membranes were isolated after the incubation. The erythrocyte membranes’ isolation was carried out using the Dodge et al. (1963) method with some modifications [34]. After incubation, RBCs were centrifuged (3000 rpm, 5 min.; 4 °C) and the cells were suspended in the membrane isolation buffer (20 mM Tris-HCl buffer (pH 7.4) with 1 mM ethylenediaminetetraacetic acid iron(III) sodium salt (EDTA) and 0.01% phenylmethylsulfonyl fluoride (PMSF)), and incubated on ice. After 15 min RBCs were centrifuged (14,000 rpm, 5 min; 4 °C). Membranes were repeatedly washed (7–8 times) with the membrane isolation buffer, until clear structures were obtained. The isolation procedure was carried out on ice, with buffer cooled down to 4 °C. The concentration of analyzed compounds was selected on the basis of our previous studies concerning BFRs-induced hemolysis and eryptosis in human RBCs [9,11].

The use of human blood (leucocyte buffy-coat) in the investigation of the effect of flame retardants on human erythrocytes has been approved by Bioethics Committee for Scientific Investigation, University of Lodz (agreement No. 7/KBBN-UŁ/II/2015).

### 4.5. Protein Oxidation

Protein damage was assessed based on the fluorescence measurement at 335 nm after excitation at 295 nm. Fluorescent properties of proteins are associated with the presence of aromatic amino acids (mainly tryptophan) in their structure. A decrease in fluorescence of a sample results from oxidative damage to tryptophan, thus allowing for the assessment of RBC membrane proteins damage [35]. The analysis was performed in 96-well plates using a microplate reader (Cary Eclipse, Varian). The results were presented as per cent of control. 

### 4.6. Statistical Analysis 

The statistical analysis was performed using the one-way analysis of variance (ANOVA) followed by the Tukey post hoc test and presented as means ± standard deviation (SD). Differences were considered to be statistically significant at *p* < 0.05. All statistical analyses were performed using the STATISTICA software (StatSoft, Inc, Tulusa, OK, USA) and GraphPad Prism software data analysis program (GraphPad Software, San Diego, CA, USA).

## 5. Conclusions

It was found that the structure of BFRs differing in the number of aromatic rings and the number of bromine atoms in the ring significantly affected their interaction with proteins.

Bromophenols quenched tryptophan fluorescence in HSA linearly over the entire range of their analyzed concentrations (0.1–10 µM). The action of bromobisphenols was more complex and at their lower concentrations TBBPS had no effect on HSA fluorescensce up to 2.5 μM. 

Among analyzed bromophenols, PBP showed the strongest oxidative effect on RBC membrane (biological model), and comparable effect to 2,4,6-TBP in case of interactions with HSA (chemical model). However, probably due to its structure, PBP did not penetrate nor disturbed the spatial structure of HSA. On the contrary, 2,4-DBP had the weakest oxidative effect among analyzed bromophenols, causing the most pronounced loss of the alpha-helix structure in HSA. 

Of all analyzed BFRs, TBBPA caused the strongest oxidative changes in RBC membrane proteins, and of the model HSA protein, causing reduction of fluorescence of tryptophan contained in them. This compound also changed albumin conformation properties, leading to impairment of the α-helix and rise of the content of the ß-sheet structure. On the other hand, TBBPS had the weakest oxidative effect on proteins, because it did not alter the secondary structure of HSA. 

## Figures and Tables

**Table 1:** Quenching constants of HSA and selected BFRs at 21 °C, over the concentration range of 0.1–10 µM. ♦K_SV_ constant [µM ^−1^] for TBBPA over the concentration range of 0.1–1 µM, and for TBBPS over the concentration range of 0.1–2.5 µM.

	**Bromobisphenols**		**Bromophenols**		
	TBBPA	TBBPS	2,4-DBP	2,4,6-TBP	PBP
**KSV**	2.461 × 10^5^ ♦	0.005 × 10^5^*♦	0.519 × 10^5^	1.482 × 10^5^	1.203 × 10^5^

**Table 2:** Composition of the secondary structure of HSA exposed to BFRs studied.

	Helix	Antiparallel	Parallel	B-turn	Rndm. Coil	Total Sum
Control	70.73 ± 2.76	2.50 ± 0.26	2.83 ± 0.29	11.43 ± 0.38	13.03 ± 1.08	100.53 ± 0.76
**Concentration 25 µg/mL**						
TBBPA	64.77 ± 1.42 *	3.17 ± 0.15 *	3.47 ± 0.15 *	12.30 ± 0.20 *	15.30 ± 0.56 *	98.97 ± 0.40
TBBPS	68.10 ± 0.82	2.77 ± 0.06	3.10 ± 0.10	11.80 ± 0.10	14.10 ± 0.30	99.87 ± 0.29
2,4-DBP	68.67 ± 1.25	2.63 ± 0.15	3.00 ± 0.10	11.63 ± 0.15	13.90 ± 0.36	99.83 ± 0.50
2,4,6-TBP	72.00 ± 1.08	2.40 ± 0.10	2.70 ± 0.10	11.27 ± 0.15	12.40 ± 0.36	100.73 ± 0.32
PBP	70.37 ± 2.58	2.53 ± 0.23	2.87 ± 0.29	11.53 ± 0.32	13.17 ± 1.07	100.47 ± 0.68
**Concentration 50 µg/mL**						
TBBPA	52.30 ± 3.49 *	4.67 ± 0.49 *	4.97 ± 0.49 *	13.97 ± 0.42 *	21.03 ± 1.93 *	96.93 ± 0.25
2,4-DBP	54.53 ± 3.44 *	3.93 ± 0.38 *	4.87 ± 0.49 *	12.90 ± 0.26 *	22.60 ± 2.46 *	98.80 ± 0.46
2,4,6-TBP	65.40 ± 1.35 *	2.97 ± 0.15	3.33 ± 0.15	12.03 ± 0.15	15.33 ± 0.65 *	99.07 ± 0.25
PBP	71.70 ± 0.10	2.40 ± 0.00	2.73 ± 0.06	11.37 ± 0.06	12.57 ± 0.06	100.80 ± 0.00

## Abbreviations

2,4,6-TBP	2,4,6-Tribromophenol
2,4-DBP	2,4-Dibromophenol
BFRs	Brominated Flame Retardants
BPA	Bisphenol A
CD	Circular dichroism
DekaBDE	Decabrominated diphenylethers
DMSO	Dimethyl sulfoxide
EDTA	Ethylenediaminetetraacetic acid iron(III) sodium salt
HBCD	Hexabromocyclododecane
hNSCs	Human neural stem cells
HSA	Human serum albumin
MRE	Mean residue ellipticity
OctaBDE	Octabrominated diphenylethers
PBP	Pentabromophenol
PentaBDE	Pentabrominated diphenylethers
PMSF	Phenylmethylsulfonyl fluoride
RBCs	Erythrocytes
SH-SY5Y	Human neuroblastoma cells
TBBPA	Tetrabromobisphenol A
TBBPS	Tetrabromobisphenol S
